# Static Analysis of Laminated Composite Plates with Periodic Curvature Using Reissner–Mindlin Plate Theory

**DOI:** 10.3390/ma19112315

**Published:** 2026-05-30

**Authors:** Ozlem Vardar, Zafer Kutug, Ayse Erdolen

**Affiliations:** 1 Department of Civil Engineering, Faculty of Engineering and Natural Sciences, Istanbul Okan University, 34959 Istanbul, Turkey; 2 Department of Civil Engineering, Faculty of Civil Engineering, Yildiz Technical University, 34220 Istanbul, Turkey; kutug@yildiz.edu.tr (Z.K.); erdolen@yildiz.edu.tr (A.E.)

**Keywords:** composite materials, periodic curvature, laminated composite, plates, static analysis, Navier method, Kirchhoff–Love theory, Reissner–Mindlin theory

## Abstract

**Highlights:**

- Static analysis of laminated composite plates with periodic curvature is presented.
- Navier solution is applied within the Reissner–Mindlin plate theory.
- Effects of transverse shear deformation on structural response are examined.
- Numerical results are validated with available analytical solutions.
- Benchmark results are provided for future studies on periodically curved plates.

**Abstract:**

Fiber-reinforced laminated composite materials are widely used in engineering applications and may exhibit periodic curvatures due to technological requirements arising during manufacturing processes. Such geometric features directly influence the mechanical behavior of structural elements. Although the dynamic and stability behaviors of curved plates have been extensively investigated in the literature, studies addressing the static analysis of composite plates with periodic curvature, particularly those incorporating transverse shear deformations, remain limited. In this study, the static behavior of laminated composite plates with periodic curvature is investigated using the Navier solution method within the framework of Reissner–Mindlin plate theory. The governing equations are derived from the Continuum Theory developed by Akbarov and Guz’, and the effects of transverse shear deformations on displacements and internal forces are examined within the Reissner–Mindlin formulation. Numerical computations are carried out using MATLAB. The accuracy and convergence of the proposed approach are verified by comparing them with existing analytical solutions in the literature for rectangular homogeneous isotropic and laminated composite plates. Considering the limited number of analytical studies on the static analysis of composite plates with periodic curvature that account for shear deformations, the present study contributes to the literature by providing benchmark results for future research.

## 1. Introduction

Composite materials are widely used in modern engineering applications due to their superior mechanical and physical properties, including high strength and stiffness, excellent corrosion and wear resistance, and low weight. However, in fiber-reinforced laminated composites, periodic or local curvatures may arise due to design requirements or manufacturing processes. These curvatures can significantly influence the mechanical behavior of such materials. Consequently, the deflection, buckling, and vibration responses of composite structures under static and dynamic loads have been extensively investigated.

Akbarov and Guz’ [1] developed continuity equations that account for periodic or local curvatures in composite materials. These equations enabled mathematical difficulties to be overcome and, in some cases, addressed the lack of solutions encountered in the analysis of curved composites within the piecewise-homogeneous body model.

Based on these equations, Yahnioglu [2] investigated two- and three-dimensional boundary value problems of strip-plate elements made of composite materials with curvature and examined the stress and displacement distributions under static loading using the finite element method (FEM).

Kutug [3] studied the free vibration and buckling behavior of plates with local or periodic curvature within the framework of the Continuum Theory developed by Akbarov and Guz’ using the Galerkin method. For this purpose, the equations of motion derived from elasticity theory, together with vibration and elastic stability equations based on a third-order refined plate theory, were employed, and the stability equations were obtained under appropriate boundary conditions. Kutug [4] also investigated the effects of curvature on the natural frequencies and mode shapes of composite strip plates for different elasticity ratios and curvature values.

Akbarov and Selim [5] analyzed the buckling behavior of composite plates with periodic curvature using the FEM within the framework of the Continuum Theory developed by Akbarov and Guz’. The effects of different boundary conditions, curvature, and the elasticity ratios on the critical buckling loads were evaluated using the FEM based on three-dimensional elasticity theory.

Vardar, Kutug, and Erdolen [6] examined the static behavior of laminated composite plates with periodic curvature and analytically demonstrated the effects of curvature on deflection, stress, and bending moment distributions. In that study, the plate equations were derived within the framework of the Continuum Theory developed by Akbarov and Guz’, based on Kirchhoff–Love plate theory and solved by the Navier Method.

Carrera [7] presented a comparative study on the free vibration behavior of simply supported homogeneous and multilayered plates within the frameworks of classical and advanced theories, highlighting the applicability limits of these theories for thin and thick plates.

Li, Zhang, and Zheng [8] performed static and free vibration analyses of laminated composite plates by combining isogeometric analysis with a third-order shear deformation theory. The accuracy of the method was demonstrated through numerical examples of isotropic and laminated composite plates with various geometries and boundary conditions.

Sayyad, Shinde, and Ghugal [9] proposed a simple trigonometric shear deformation theory that does not require a shear correction factor to investigate the bending, buckling, and free vibration behavior of cross-ply composite plates. The analyses were carried out using the Navier method, and the obtained displacement, stress, critical buckling load, and natural frequency results were shown to be in good agreement with existing studies.

Nguyen, Tran, Pham, Nguyen, and Do [10] investigated the static bending and free vibration behavior of simply supported composite plates using a simplified first-order shear deformation theory. The solutions were obtained using the Navier method, and their accuracy was validated through a comparison with existing studies based on different theories. Unlike the classical Reissner–Mindlin theory, the refined first-order shear deformation theory used in this study assumes a parabolic distribution of transverse shear stresses through the plate thickness. Therefore, the theory satisfies the traction-free boundary conditions at the plate surfaces and does not require a shear correction factor.

Bacciochi and Tarantino [11] studied the displacement and stress fields of laminated fiber-reinforced composite plates within the framework of a third-order theory and obtained numerical results. The Navier method was used to solve the governing equations, and the numerical examples were presented to illustrate the effects of different plate theories, particularly for thick plates.

Determining the mechanical behavior of composite plates with periodic curvature is inherently challenging due to geometric complexities. However, accurately predicting this behavior is of great importance for advanced engineering applications.

Therefore, in the present study, the static behavior of laminated composite plates with periodic curvature is investigated within the framework of the Continuum Theory developed by Akbarov and Guz’ [1,12], based on Reissner–Mindlin plate theory and employing the Navier solution method. The results are evaluated by comparing them with previous Kirchhoff–Love plate theory results [6].

Although numerous studies exist on the mechanical behavior of homogeneous isotropic and laminated composite plates, studies addressing the static analysis of composite plates with periodic curvature—particularly those focusing on deflection, bending moments, and stress distributions—remain limited. In this study, the effects of material curvature on the static behavior are examined in detail within the framework of the Reissner–Mindlin plate theory, with the aim of addressing the research gap identified by Akbarov and Guz’ [13]. The analyses of homogeneous isotropic plates, laminated composite plates, and laminated plates with periodic curvature are carried out using MATLAB 2024a codes. The results for homogeneous isotropic and laminated composite plates are validated against the available literature results, confirming the accuracy of the derived equations and the developed computational model. Therefore, the validated numerical framework is employed in the analyses of laminated plates with periodic curvature.

The literature demonstrates that the normalized effective material constants of composite materials depend on the geometric configurations, volume fractions, and elastic properties of their constituent phases [14].

Reissner [15] developed a bending theory that accounts for transverse shear deformations, in contrast to the classical thin plate theory. In this theory, a more general system of differential equations is obtained, allowing the definition of three independent boundary conditions along the plate edges. This approach eliminates the corner-force singularities predicted by classical plate theory in certain problems and yields more realistic results in cases where thickness effects cannot be neglected.

Mindlin [16] derived a two-dimensional formulation for the bending behavior of isotropic elastic plates from three-dimensional elasticity equations, incorporating the effects of rotary inertia and transverse shear deformation. Similar to Timoshenko beam theory, this formulation accounts for shear deformations and provides improved accuracy for moderately thick plates.

Finally, the theoretical background and modeling approaches adopted in this study are based on classical references commonly used in the analysis of composite plates and structural mechanics [12,14,17,18].

## 2. Materials and Methods

**Figure 1:**
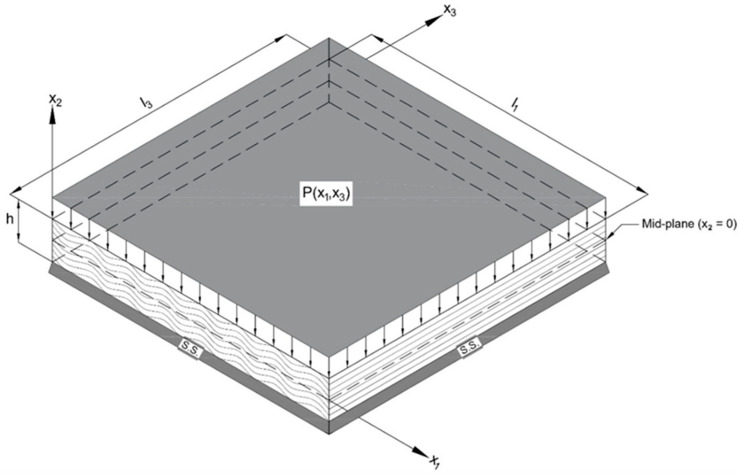
Geometry and coordinate system of the composite plate with periodic curvature.

A simply supported (S.S.) rectangular plate is considered. The thickness direction is defined along the $x_{2}$ axis, with bounds $-h/2\;\leq \;x_{2}\;\leq \;h/2$. The in-plane dimensions are defined along the $x_{1}$ and $x_{3}$ axes, with lengths $l_{2}$ and ${\;l}_{3}$, respectively (Figure 1).

According to Reissner–Mindlin plate theory, the displacement components of the plate are expressed as:

(1) $$u_{1}\left(x_{1},x_{2}{,x}_{3}\right)=u\left(x_{1},x_{3}\right)+x_{2}\psi_{1}\left(x_{1},x_{3}\right)$$

(2) $$u_{2}\left(x_{1},x_{2},x_{3}\right)=w\left(x_{1},x_{3}\right)$$

(3) $$u_{3}\left(x_{1},x_{2}{,x}_{3}\right)=v\left(x_{1},x_{3}\right)+x_{2}\psi_{3}\left(x_{1},x_{3}\right)$$

where $u_{1},\;u_{2}$ and $u_{3}$ denote the displacement components in the $x_{1},\;x_{2}$ and $x_{3}$ directions, respectively. The quantities u, w and v represent the displacements of the mid-plane of the plate. The parameters $\psi_{1}$ and $\psi_{3}$ denote the rotations of cross-sections parallel to the $x_{1}$ and $x_{3}$ axes, respectively.

**Figure 2:**
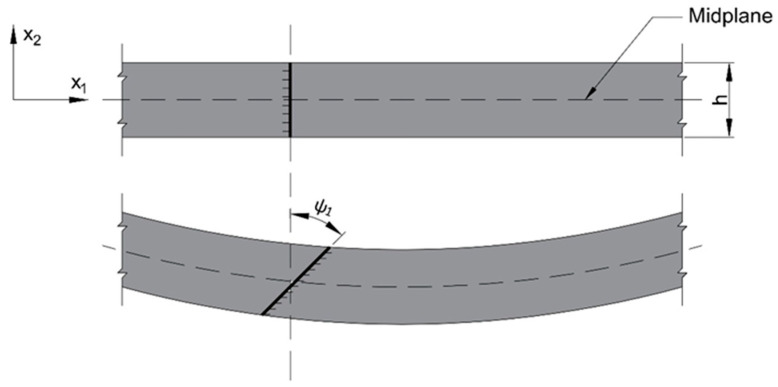
Cross-sectional behavior before and after deformation according to Reissner–Mindlin plate theory.

In Reissner–Mindlin plate theory, line elements initially normal to the mid-plane are assumed to remain straight but not necessarily perpendicular to the mid-plane after deformation [18]. Within this framework, the rotations of cross-sections through the plate thickness are taken into account (Figure 2). In Figure 2, the rotation $\psi_{1}$ is illustrated schematically; similarly, the rotation $\psi_{3}$ is defined for the other in-plane direction.

The stress–strain relationship is given by:

(4) $$\sigma_{ij}=C_{ijkl}\varepsilon_{kl}\;i,j,k,l=1,2,3.$$

The strain–displacement relationship is expressed as:

(5) $$\varepsilon_{ij}=\frac{1}{2}\left(\frac{\partial u_{i}}{{\partial x}_{j}}+\frac{\partial u_{j}}{\partial x_{i}}\right)\;i,j=1,2,3.$$

For composite plates, the stress–strain relation given in Equation (4) can be expressed in the following form:

(6) $$\sigma_{i}=A_{ij}\varepsilon_{j}\;i,j=1,2,3,4,5,6.$$

The expanded form of Equation (6) is given below:

(7) $$\left[\begin{array}{c} \sigma_{1}\\ \sigma_{2}\\ \sigma_{3}\\ \sigma_{4}\\ \sigma_{5}\\ \sigma_{6} \end{array}\right]=\left[\begin{array}{c} A_{11}\;\;\;\\ A_{21}\;\;\;\\ A_{31}\;\;\\ A_{41}\;\;\\ A_{51}\;\;\\ A_{61}\;\; \end{array}\begin{array}{c} A_{12}\;\\ A_{22}\;\\ A_{32}\\ A_{42}\\ A_{52}\\ A_{62} \end{array}\begin{array}{c} A_{13}\;\;\;\\ A_{23}\\ A_{33}\\ A_{43}\\ A_{53}\\ A_{63} \end{array}\begin{array}{c} A_{14}\;\;\;\\ A_{24}\;\\ A_{34}\\ A_{44}\\ A_{54}\\ A_{64} \end{array}\begin{array}{c} A_{15}\;\;\;\\ A_{25}\;\\ A_{35}\\ A_{45}\\ A_{55}\\ A_{65} \end{array}\begin{array}{c} A_{16}\\ A_{26}\\ A_{36}\\ A_{46}\\ A_{56}\\ A_{66} \end{array}\right]\left[\begin{array}{c} \varepsilon_{1}\\ \varepsilon_{2}\\ \varepsilon_{3}\\ \varepsilon_{4}\\ \varepsilon_{5}\\ \varepsilon_{6} \end{array}\right]$$

where the stress and strain components are defined, respectively, as

(8) $$\left[\begin{array}{c} \sigma_{1}\\ \sigma_{2}\\ \sigma_{3}\\ \sigma_{4}\\ \sigma_{5}\\ \sigma_{6} \end{array}\right]=\left[\begin{array}{c} \sigma_{11}\\ \sigma_{22}\\ \sigma_{33}\\ \sigma_{23}\\ \sigma_{13}\\ \sigma_{12} \end{array}\right]\;,\;\left[\begin{array}{c} \varepsilon_{1}\\ \varepsilon_{2}\\ \varepsilon_{3}\\ \varepsilon_{4}\\ \varepsilon_{5}\\ \varepsilon_{6} \end{array}\right]=\left[\begin{array}{c} \varepsilon_{11}\\ \varepsilon_{22}\\ \varepsilon_{33}\\ \varepsilon_{23}\\ \varepsilon_{13}\\ \varepsilon_{12} \end{array}\right]$$

According to the assumptions of the adopted plate formulation, $\varepsilon_{22}=0.\;$Substituting Equations (1)–(3) into Equation (5) and Equation (7), the following expression is obtained:

(9) $$\left[\begin{array}{c} \sigma_{1}\\ \sigma_{3}\\ \sigma_{4}\\ \sigma_{5}\\ \sigma_{6} \end{array}\right]=\left[\begin{array}{ccccc} A_{11} & A_{13} & 0 & 0 & A_{16}\\ A_{31} & A_{33} & 0 & 0 & A_{36}\\ 0 & 0 & A_{44} & A_{45} & 0\\ 0 & 0 & A_{54} & A_{55} & 0\\ A_{61} & A_{63} & 0 & 0 & A_{66} \end{array}\right]\left[\begin{array}{c} u_{,1}+x_{2}\psi_{1,1}\\ v_{,3}+x_{2}\psi_{3,3}\\ w_{,3}+\psi_{3}\\ \left(u_{,3}+v_{,1}+x_{2}\left(\psi_{1,3}+\psi_{3,1}\right)\right)\\ w_{,1}+\psi_{1} \end{array}\right]$$

where the curvature exists only in the $x_{1}$ direction, the material coefficients depend on $x_{1}$ [2].

$$A_{11}\left(x_{1}\right)=A_{11}^{0}\phi_{1}^{4}\left(x_{1}\right)+2A_{12}^{0}\phi_{1}^{2}\left(x_{1}\right)\phi_{2}^{2}\left(x_{1}\right)+A_{22}^{0}\phi_{2}^{4}(x_{1})+4A_{66}^{0}\phi_{1}^{2}(x_{1})\phi_{2}^{2}(x_{1})$$

$$A_{13}(x_{1})=A_{13}^{0}\phi_{1}^{2}(x_{1})+A_{23}^{0}\phi_{2}^{2}(x_{1})$$

$$A_{16}\left(x_{1}\right)=\left(A_{12}^{0}-A_{11}^{0}+2A_{66}^{0}\right)\phi_{1}^{3}\left(x_{1}\right)\phi_{2}\left(x_{1}\right)+\left(A_{12}^{0}-A_{11}^{0}+2A_{66}^{0}\right)\phi_{1}\left(x_{1}\right)\phi_{2}^{3}\left(x_{1}\right)$$

$$A_{33}(x_{1})=A_{33}^{0}$$

(10) $$A_{36}\left(x_{1}\right)=\left(A_{23}^{0}-A_{13}^{0}\right)\phi_{1}\left(x_{1}\right)\phi_{2}\left(x_{1}\right)$$

$$A_{44}(x_{1})=A_{44}^{0}\phi_{1}^{2}(x_{1})+A_{55}^{0}\phi_{2}^{2}(x_{1})$$

$$A_{45}\left(x_{1}\right)=\left(A_{44}^{0}-A_{55}^{0}\right)\phi_{1}\left(x_{1}\right)\phi_{2}\left(x_{1}\right)$$

$$A_{55}(x_{1})=A_{55}^{0}\phi_{1}^{2}(x_{1})+A_{44}^{0}\phi_{2}^{2}(x_{1})$$

$$A_{66}\left(x_{1}\right)=\left(A_{11}^{0}-{2A}_{12}^{0}+A_{22}^{0}\right)\phi_{1}^{2}\left(x_{1}\right)\phi_{2}^{2}\left(x_{1}\right)+A_{66}^{0}(\phi_{1}^{4}\left(x_{1}\right)+\phi_{2}^{4}\left(x_{1}\right)-2\phi_{1}^{2}\left(x_{1}\right)\phi_{2}^{2}\left(x_{1}\right))$$

where the auxiliary curvature-related functions $\phi_{1}$ and $\phi_{2}$ are explicitly defined in Equations (12) and (13). $A_{11}^{0},\;A_{12}^{0}{,\;A}_{22}^{0},\;A_{66}^{0},\;{{A_{13}^{0},\;A}_{23}^{0},\;A}_{33}^{0},\;A_{55}^{0}$ and $A_{44}^{0}$, are normalized material constants of composite layers without curvature, defined as follows:

$$A_{11}^{0}=A_{33}^{0}=\mu_{1}\eta_{1}+\mu_{2}\eta_{2}+\left(\mu_{1}+\eta_{1}\right)\eta_{1}+\left(\mu_{2}+\eta_{2}\right)\eta_{2}-\frac{{\left(\lambda_{1}-\lambda_{2}\right)}^{2}}{\left(\lambda_{1}+{2\mu }_{1}\right)\eta_{2}+\left(\lambda_{2}+{2\mu }_{2}\right)\eta_{1}}$$

$$A_{12}^{0}=A_{23}^{0}=\lambda_{1}\eta_{1}+\lambda_{2}\eta_{2}-(\lambda_{1}-\lambda_{2})\eta_{1}\eta_{2}\frac{\left(\lambda_{1}+2\mu_{1})(\lambda_{2}+2\mu_{2}\right)}{\left(\lambda_{1}+{2\mu }_{1}\right)\eta_{2}+\left(\lambda_{2}+{2\mu }_{2}\right)\eta_{1}}$$

$$A_{22}^{0}=\left(\lambda_{1}+2\mu_{1}\right)\eta_{1}+\left(\lambda_{2}+2\mu_{2}\right)\eta_{2}-\eta_{1}\eta_{2}\frac{{\left[\left(\lambda_{1}+2\mu_{1}\right)-(\lambda_{2}+2\mu_{2})\right]}^{2}}{\left(\lambda_{1}+{2\mu }_{1}\right)\eta_{2}+\left(\lambda_{2}+{2\mu }_{2}\right)\eta_{1}}$$

(11) $$A_{66}^{0}=A_{55}^{0}=\frac{\mu_{1}\mu_{2}}{\mu_{1}\eta_{2}+\mu_{2}\eta_{1}}$$

$$A_{13}^{0}=\left(\lambda_{1}+\mu_{1}\right)\eta_{1}+\left(\lambda_{2}+\mu_{2}\right)\eta_{2}-\eta_{1}\eta_{2}\frac{{\left(\lambda_{1}-\lambda_{2}\right)}^{2}}{\left(\lambda_{1}+2\mu_{1}\right)\eta_{2}+\left(\lambda_{2}+2\mu_{2}\right)\eta_{1}}-\eta_{1}\mu_{1}-\eta_{2}\mu_{2}$$

$$A_{44}^{0}{=\mu }_{1}\eta_{1}+\mu_{2}\eta_{2}$$

where $\mu_{1},\mu_{2}$ and $\eta_{1},\eta_{2}$ are the Lamé constants of the matrix and reinforcement materials, respectively.

The curvature function parameters $\phi_{1}$ and $\phi_{2}$ are defined as follows:

(12) $$\begin{array}{c} \phi_{1}=\frac{1}{\sqrt{1+{\frac{dF\left(x_{1}\right)}{dx_{1}}}^{2}}}\\ \phi_{2}=\phi_{1}\frac{dF\left(x_{1}\right)}{dx_{1}} \end{array}$$

where

(13) $$\begin{array}{c} F\left(x_{1}\right)=\varepsilon f(x_{1})\\ \varepsilon =\frac{\bar{h}}{\Lambda },\;f\left(x_{1}\right)=\sin\left(\frac{\pi x_{1}}{\Lambda }+\delta \right) \end{array}$$

**Figure 3:**
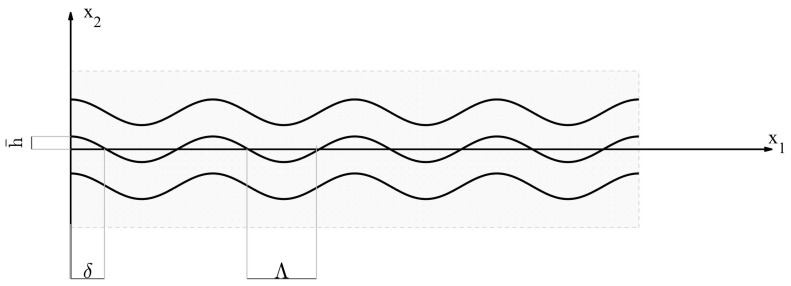
Plate with periodic curvature showing the geometric parameters h, Λ and δ.

In Figure 3, Λ, h and δ denote the wavelength, amplitude, and phase shift in the curvature, respectively.

The equilibrium equations of elasticity,

(14) $$\sigma_{ij,j}+F_{i}=0\;i,j=1,2,3.$$

By substituting Equation (9) into Equation (14), the stress components are first expressed in terms of the displacement and rotational variables. Subsequently, the internal force and bending moment components are obtained by integrating the stress components through the plate thickness. These quantities are then substituted into the governing equilibrium equations of the plate, leading to the coupled governing equations given in Equations (15)–(19).

(15) $$\begin{array}{c} A_{11,1}hu_{,1}+A_{11}hu_{,11}+A_{13,1}hv_{,3}+A_{13}hv_{,31}+A_{16,1}hw_{,1}+A_{16}hw_{,11}+A_{16,1}h\psi_{1}\\ +A_{16}h\psi_{1,1}+A_{45}hw_{,33}+A_{45}h\psi_{3,3}+A_{55}hu_{,33}+A_{55}{hv}_{,13}=0 \end{array}$$

(16) $$\begin{array}{c} A_{16,1}hu_{,1}+A_{16}hu_{,11}+A_{36,1}hv_{,3}+A_{36}hv_{,31}+A_{66,1}hw_{,1}+A_{66}hw_{,11}+A_{66,1}h\psi_{1}\\ +A_{66}h\psi_{1,1}+A_{44}hw_{,33}+A_{44}h\psi_{3,3}+A_{45}hu_{,33}+A_{45}hv_{,13}=P\left(x_{1},x_{3}\right) \end{array}$$

(17) $$\begin{array}{c} A_{45,1}hw_{,3}+A_{45}hw_{,31}+A_{45,1}h\psi_{3}+A_{45}h\psi_{3,1}+A_{55,1}hu_{,3}+A_{55}hu_{,31}+A_{55,1}hv_{,1}\\ +A_{55}hv_{,11}+A_{13}hu_{,13}+A_{33}hv_{,33}+A_{36}hw_{,13}+A_{36}h\psi_{1,3}=0 \end{array}$$

(18) $$\begin{array}{c} A_{11,1}\frac{h^{3}}{12}\psi_{1,1}+A_{11}\frac{h^{3}}{12}\psi_{1,11}+A_{13,1}\frac{h^{3}}{12}\psi_{3,3}+A_{13}\frac{h^{3}}{12}\psi_{3,31}+A_{55}\frac{h^{3}}{12}\psi_{1,33}\\ +A_{55}\frac{h^{3}}{12}\psi_{3,13}=A_{16}hu_{,1}+A_{36}hv_{,3}+A_{66}hw_{,1}+A_{66}h\psi_{1} \end{array}$$

(19) $$\begin{array}{c} h_{55,1}\frac{h^{3}}{12}\psi_{1,3}+A_{55}\frac{h^{3}}{12}\psi_{1,31}+A_{551}\frac{h^{3}}{12}\psi_{3,1}+A_{55}\frac{h^{3}}{12}\psi_{3,11}+A_{13}\frac{h^{3}}{12}\psi_{1,13}\\ +A_{33}\frac{h^{3}}{12}\psi_{3,33}=A_{44}hw_{,3}+A_{44}h\psi_{3}+A_{45}hu_{,3}+A_{45}hv_{,1} \end{array}$$

The derived equilibrium equations (Equations (15)–(19)) involve five unknowns, namely the displacements u, v, w and the rotational components $\psi_{1}$ and $\psi_{3}$. Unlike the single equilibrium equation used in classical thin plate theories, these five equations simultaneously account for in-plane effects and rotational components. Consequently, the static behavior of relatively thick composite plates with periodic curvature can be more accurately and realistically represented.

For a plate simply supported along all four edges, the boundary conditions are defined in terms of displacement and moment components. These boundary conditions enable the application of a Navier-type solution:

(20) $$\begin{array}{c} w\left(0,x_{3}\right)=w\left(l_{1},x_{3}\right)=0,\;M_{11}\left(0,x_{3}\right)=M_{11}\left(l_{1},x_{3}\right)=0\\ w\left(x_{1},0\right)=w\left(x_{1},l_{3}\right)=0,\;M_{33}\left(x_{1},0\right)=M_{33}\left(x_{1},l_{3}\right)=0. \end{array}$$

The displacement fields satisfying these boundary conditions are expressed as follows:

(21) $$u(x_{1},x_{3})=\sum_{m=1}^{\infty }\sum_{n=1}^{\infty }u_{mn}\cos\frac{m\pi x_{1}}{l_{1}}\sin\frac{n\pi x_{3}}{l_{3}}$$

(22) $$v(x_{1},x_{3})=\sum_{m=1}^{\infty }\sum_{n=1}^{\infty }v_{mn}\sin\frac{m\pi x_{1}}{l_{1}}\cos\frac{n\pi x_{3}}{l_{3}}$$

(23) $$w(x_{1},x_{3})=\sum_{m=1}^{\infty }\sum_{n=1}^{\infty }w_{mn}\sin\frac{m\pi x_{1}}{l_{1}}\sin\frac{n{\pi x}_{3}}{l_{3}}$$

(24) $$\psi_{1}(x_{1},x_{3})=\sum_{m=1}^{\infty }\sum_{n=1}^{\infty }\psi_{1mn}\cos\frac{m\pi x_{1}}{l_{1}}\sin\frac{n\pi x_{3}}{l_{3}}$$

(25) $$\psi_{3}(x_{1},x_{3})=\sum_{m=1}^{\infty }\sum_{n=1}^{\infty }\psi_{3mn}\sin\frac{m\pi x_{1}}{l_{1}}\cos\frac{n\pi x_{3}}{l_{3}}$$

According to the Navier method, the distributed load acting on the plate $P(x_{1},x_{3})$ is expressed by the following series expansion [18]

(26) $$P(x_{1},x_{3})=\sum_{m=1}^{\infty }\sum_{n=1}^{\infty }P_{mn}\sin\frac{m\pi x_{1}}{l_{1}}\sin\frac{n\pi x_{3}}{l_{3}}$$

For a uniformly distributed load, the coefficients $P_{mn}$ are defined as follows [18]

(27) $$P_{mn}=\frac{16P_{o}}{\pi^{2}mn}\;m,n=1,3,5\ldots$$

By substituting Equations (21)–(25) into the corresponding terms of Equations (15)–(19), the governing equations are transformed into a system expressed in terms of the Fourier coefficients. In the numerical implementation, the infinite Fourier series expansions are truncated to a finite number of terms, leading to a finite-dimensional approximation space within the Galerkin framework. The number of Fourier terms is selected according to the convergence behavior of the numerical results, as verified through convergence analyses, and different truncation levels are examined until converged solutions are obtained.

Within the Galerkin procedure, the globally defined trigonometric functions used in the displacement and load expansions are also employed as weighting functions. Owing to the orthogonality properties of the sine and cosine functions, only the corresponding modal terms remain in the final system of equations. The resulting weighted residual equations are integrated over the solution domain using the Gauss–Legendre integration method, and five equilibrium equations corresponding to the unknown Fourier coefficients are obtained. The detailed derivation steps and the explicit forms of these equations are presented in Ref. [19].

## 3. Results

For the simply supported plate on all four edges, with loading and boundary conditions shown in Figure 1, the plate dimensions used in the numerical analyses are taken as $l_{1}=1\;m$, $l_{3}=2\;m$ and thickness h=0.1 m. The applied load is defined as a uniformly distributed load given by $P\left(x_{1},x_{3}\right)=10,000\;N/m^{2}$.

The geometric and loading parameters are kept constant to enable a direct comparison with the results obtained within the framework of the Kirchhoff–Love theory [19]. This approach allows the effect of the theoretical model differences on deflection, moment, and stress distributions to be evaluated independently of other variables.

Numerical analyses are performed for three cases: (i) homogeneous isotropic material, (ii) laminated composite material, and (iii) laminated composite material with periodic curvature. For the laminated composite case, Young’s modulus $E_{1}=20\;GPa$ and Poisson’s ratio $\nu_{1}=0.3$ are used for the matrix material, while Young’s modulus $E_{2}=200\;GPa$ and Poisson’s ratio $\nu_{2}=0.3$ are used for the reinforcement material. The volume fractions for the last two phases are $\eta_{1}=\eta_{2}=0.5.$ For the homogeneous isotropic plate, only the mechanical properties of the matrix phase are used for comparison purposes.

In addition, the analyses are not restricted to a single geometric configuration. To investigate the effect of the plate aspect ratio on structural behavior, solutions are obtained for $l_{1}/l_{3}=0.1,0.5,1.0,2.0.$ In this context, $l_{1}$ is kept constant, while different geometric ratios are achieved by varying $l_{3}$. Thus, the influence of the aspect ratio on the deflection, moment, and stress distributions is also evaluated.

The deflection, moment, and stress results obtained for homogeneous isotropic and laminated composite plates are compared with those reported in the literature within the framework of Kirchhoff–Love theory [19]. The relative errors $\left(e_{r}\right)$ between the two theories are presented in the relevant tables (Table 1 and Table 2).

The deflection values at the midpoint of the plate, based on the Reissner–Mindlin theory, are presented in Table 3. Under the same conditions, the absolute values of the bending moment components, $\left|M_{11}\right|$ and $\left|M_{33}\right|$, are given in Table 4 and Table 5, respectively. In addition, to illustrate the stress distribution within the plate, the absolute values of the normal and shear stress components $\left(\left|\sigma_{11}\right|,\left|\sigma_{33}\right|,\left|\sigma_{23}\right|,\left|\sigma_{13}\right|,\left|\sigma_{12}\right|\right)$ are presented in Table 5.

**Figure 4:**
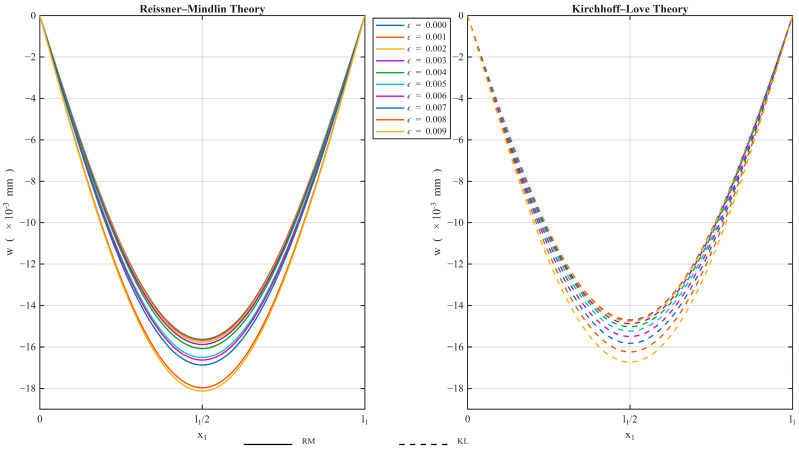
Deflection of the laminated composite plate with periodic curvature as a function of ε for $l_{1}/l_{3}=0.1\;$and $E_{2}/E_{1}=10$, based on Reissner–Mindlin theory.

**Figure 5:**
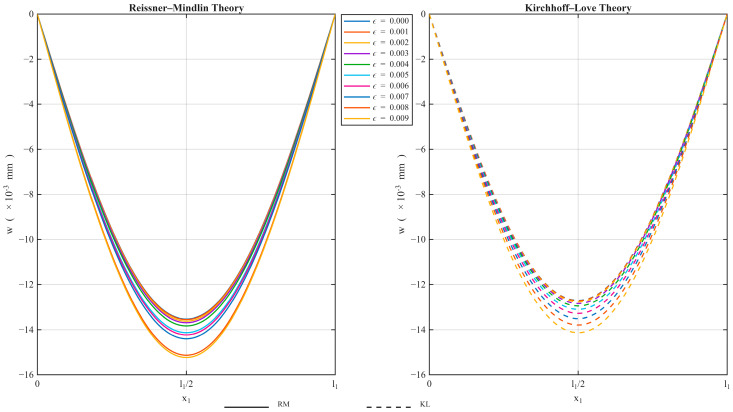
Deflection of the laminated composite plate with periodic curvature as a function of ε for $l_{1}/l_{3}=0.5\;$and${\;E}_{2}/E_{1}=10$, based on Reissner–Mindlin theory.

**Figure 6:**
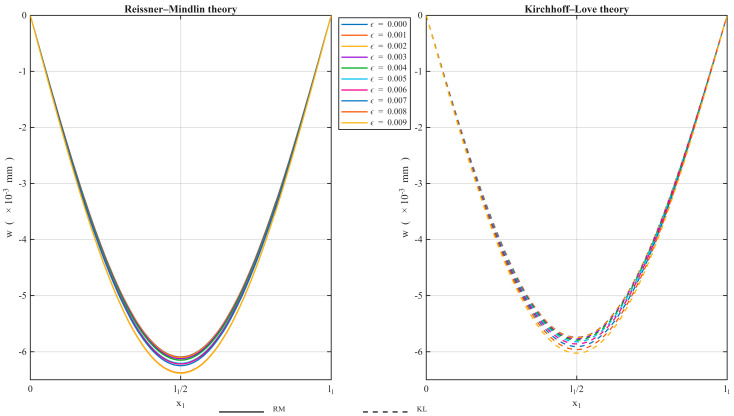
Deflection of the laminated composite plate with periodic curvature as a function of ε for $l_{1}/l_{3}=1.0\;$and ${\;E}_{2}/E_{1}=10$, based on Reissner–Mindlin theory.

**Figure 7:**
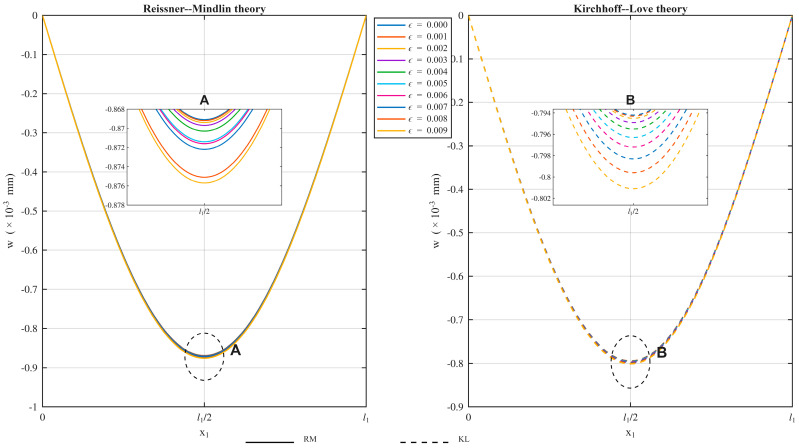
Deflection of the laminated composite plate with periodic curvature as a function of ε for $l_{1}/l_{3}=2.0\;$and $E_{2}/E_{1}=10$, based on Reissner–Mindlin theory.

The deflection of the plate at the midpoint, obtained based on the Reissner–Mindlin theory for different values of ε and aspect ratios, is presented in Figure 4, Figure 5, Figure 6 and Figure 7.

**Figure 8:**
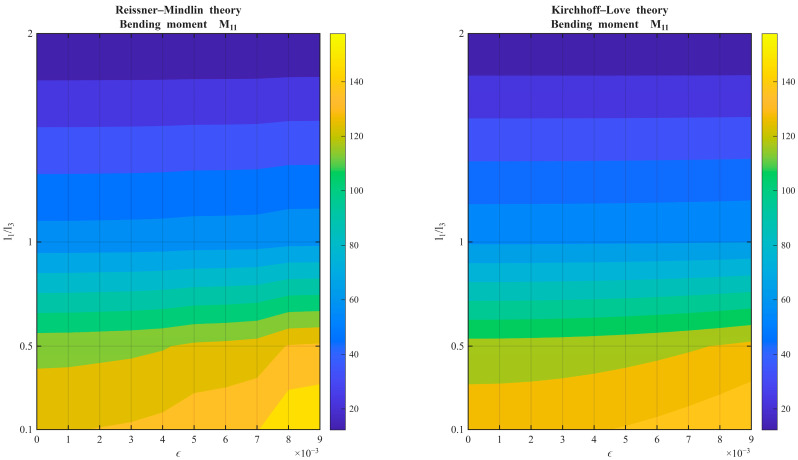
Midpoint bending moment component $M_{11}$ of the laminated composite plate with periodic curvature as functions of ε and $l_{1}/l_{3}$ for $E_{2}/E_{1}=10$, based on Reissner–Mindlin theory and compared with Kirchhoff–Love theory (×10^−2^ kN·m).

**Figure 9:**
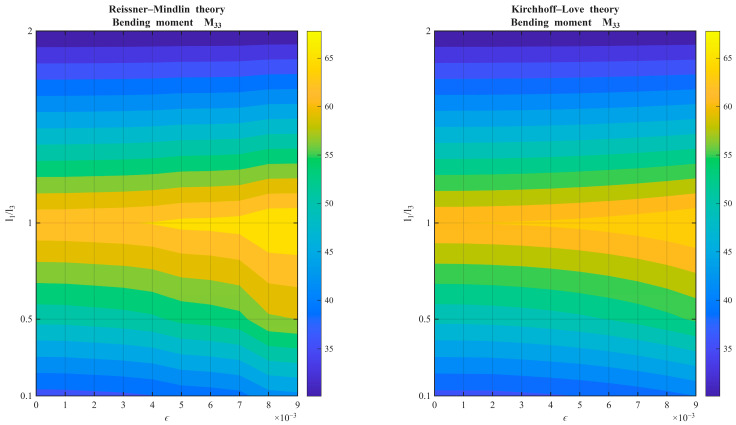
Midpoint bending moment component $M_{33}$ of the laminated composite plate with periodic curvature as functions of ε and $l_{1}/l_{3}$ for $E_{2}/E_{1}=10$, based on Reissner–Mindlin theory and compared with Kirchhoff–Love theory (×10^−2^ kN·m).

The variation in the bending moment components for different values of ε, and the span ratios are presented in Figure 8 and Figure 9.

**Figure 10:**
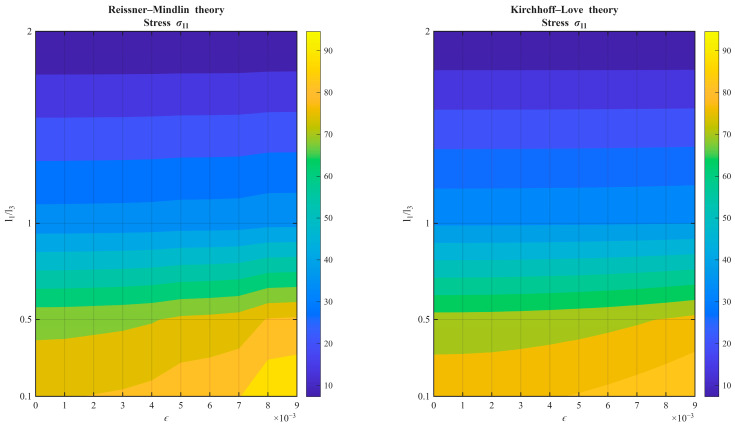
$\sigma_{11}$ stress variation in the composite plate with periodic curvature versus ε and $l_{1}/l_{3}$ ${(E}_{2}/E_{1}=10)$ based on the Reissner–Mindlin theory, compared with the Kirchhoff–Love theory (×10^−2^ MPa), at $\left(l_{1}/2,l_{3}/2,-h/2\right).$

**Figure 11:**
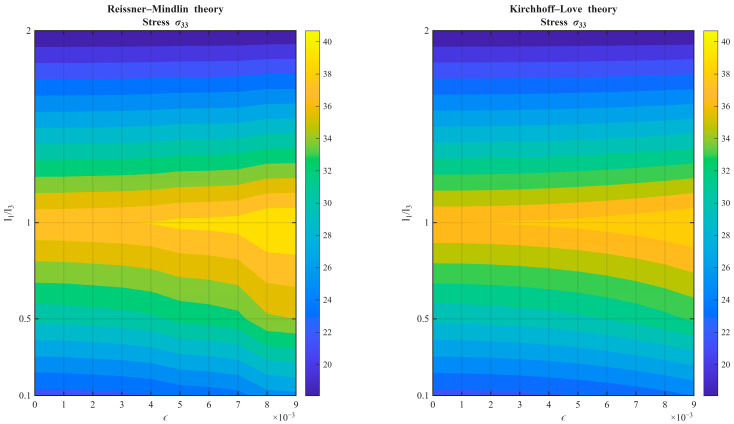
$\sigma_{33}$ stress variation in the composite plate with periodic curvature versus ε and $l_{1}/l_{3}$ $(E_{2}/E_{1}=10)$ based on the Reissner–Mindlin theory, compared with the Kirchhoff–Love theory (×10^−2^ MPa), at $\left(l_{1}/2,l_{3}/2,-h/2\right).$

**Figure 12:**
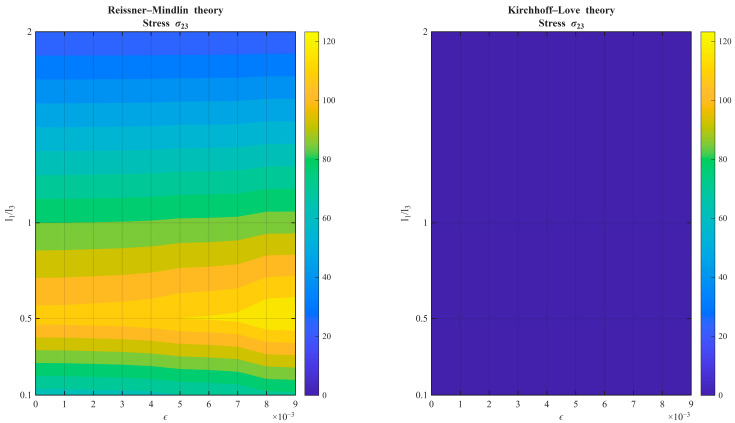
$\sigma_{23}$ stress variation in the composite plate with periodic curvature versus ε and $l_{1}/l_{3}$ ${(E}_{2}/E_{1}=10)\;$based on the Reissner–Mindlin theory, compared with the Kirchhoff–Love theory (×10^−2^ MPa), at $\left(l_{1}/2,l_{3},0\right).$

**Figure 13:**
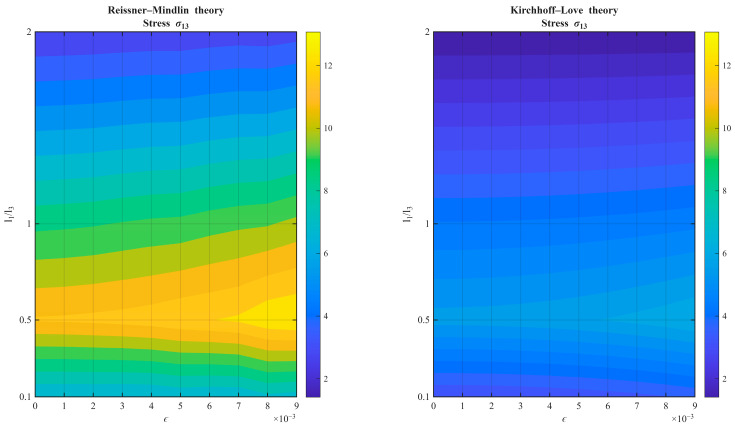
$\sigma_{13}$ stress variation in the composite plate with periodic curvature versus ε and $l_{1}/l_{3}$ ${(E}_{2}/E_{1}=10)$ based on the Reissner–Mindlin theory, compared with the Kirchhoff–Love theory (×10^−2^ MPa), at $\left(l_{1},l_{3},0\right)$.

**Figure 14:**
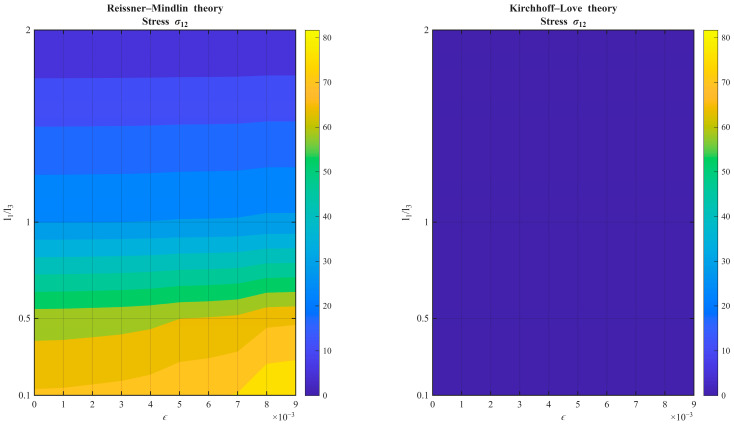
$\sigma_{12}$ stress variation in the composite plate with periodic curvature versus ε and $l_{1}/l_{3}$ ${(E}_{2}/E_{1}=10)\;$based on the Reissner–Mindlin theory, compared with the Kirchhoff–Love theory (×10^−2^ MPa), at $\left(l_{1},l_{3}/2,\;0\right)$.

Furthermore, the distributions of normal and shear stresses in the plate, evaluated for various ε values and span ratios based on the Reissner–Mindlin and Kirchhoff–Love theories, are illustrated in Figure 10, Figure 11, Figure 12, Figure 13 and Figure 14.

## 4. Discussion

In order to better understand the structural behavior reflected in the obtained results, the following discussion is presented.

For homogeneous isotropic rectangular plates, the relative difference between the deflection values obtained using the Navier method based on Kirchhoff–Love plate theory and those calculated using the Reissner–Mindlin plate theory is found to be 2.64%. For laminated composite plates, this difference increases to 6.06%. In contrast, the moment and stress values are observed to be in close agreement between the two theories.

These results indicate that shear deformation plays a more significant role in deflection behavior, particularly in composite plates. Accordingly, the static analysis of laminated composite plates with periodic curvature in a single direction is carried out using the Reissner–Mindlin plate theory for different curvature parameters  (ε=0.001−0.009). In this way, transverse shear stresses and through-thickness stress components, which cannot be obtained within the framework of Kirchhoff–Love theory, are determined.

Kirchhoff–Love theory can only represent bending-induced normal stresses and neglects transverse shear deformations. In contrast, Reissner–Mindlin theory accounts for shear effects through the thickness, enabling a more comprehensive stress distribution.

The overall implications of these results are further addressed in the Conclusion section.

## 5. Conclusions

In this study, the behavior of a composite plate with periodic curvature has been investigated using the Reissner–Mindlin (RM) and Kirchhoff–Love (KL) plate theories. Deflection, moment, and stress components have been comparatively evaluated for different values of the curvature parameter (ε) and aspect ratio $\left(l_{1}/l_{3}\right)$. The main results are summarized as follows:

1. As the curvature parameter ε increases, the deflection at the plate midpoint increases for all $l_{1}/l_{3}$ ratios. In all examined cases, the Reissner–Mindlin theory yields deflection values approximately 5–9% higher than those of the Kirchhoff–Love theory.
2. The difference in deflection between the RM and KL theories becomes more pronounced, particularly at smaller $l_{1}/l_{3}$ ratios and higher ε values. This indicates that shear deformation effects become more significant within these ranges.
3. As the curvature parameter increases, the bending moment components $M_{11}$ and $M_{33}$ at the plate midpoint generally increase. The $M_{11}$ moment values obtained from the Reissner–Mindlin theory are approximately 1–7% higher than those obtained by the Kirchhoff–Love theory. In contrast, although the differences in $M_{33}$ are generally small, they can reach up to approximately 7%, as the aspect ratio $l_{1}/l_{3}$ increases, causing the plate to behave like a strip.
4. Regarding the normal stress components $\sigma_{11}\;$and $\sigma_{33}$, the differences between the RM and KL theories are generally limited, ranging from approximately 0% to 7%.
5. The most significant difference between the two theories is observed in the stress components associated with transverse shear effects. While the Reissner–Mindlin theory produces non-zero values for $\sigma_{23}$ and$\;\sigma_{12}$, these stress components are inherently zero in the Kirchhoff–Love theory. This clearly demonstrates the limitation of the classical plate theory in representing shear deformation.
6. For the $\sigma_{13}$ stress component, the Reissner–Mindlin theory predicts values approximately 47–55% higher than those obtained using the Kirchhoff–Love theory. This result indicates that shear deformation plays a significant role in the stress distribution of composite plates with periodic curvature.
7. The results show that the Kirchhoff–Love theory can provide acceptable results for thin plates and low curvature parameters. However, when curvature effects increase and shear stresses become significant, the Reissner–Mindlin theory yields more realistic results.

In conclusion, this study presents a comprehensive comparative evaluation of composite plates with periodic curvature, not only in terms of deflection and moment components but also of different stress components, thereby providing a more detailed assessment than the existing literature.

## Figures and Tables

**Table 1:** Deflection, moment, and stress values at the midpoint of the isotropic plate $l_{1}/l_{3}=\;0.5.$

	[19]	Reissner–Mindlin	Relative Errors $e_{r}$
w (mm)	0.05536	0.05686	% 2.64
$M_{11}$ (kN·m)	1.032	1.022	% 0.98
$M_{33}$ (kN·m)	0.474	0.474	% 0.00
$\sigma_{11}$ (MPa) $(x_{2}=\;-h/2$)	0.613	0.613	% 0.00
$\sigma_{33}$ (MPa) $(x_{2}=\;-h/2$)	0.284	0.284	% 0.00

**Table 2:** Deflection, moment, and stress values at the midpoint of the laminated composite plate $l_{1}/l_{3}=\;0.5$.

	[19]	Reissner–Mindlin	Relative Errors $e_{r}$
w (mm)	0.01271	0.01353	% 6.06
$M_{11}$ (kN·m)	1.207	1.203	% 0.33
$M_{33}$ (kN·m)	0.501	0.513	% 0.23
$\sigma_{11}$ (MPa) ${(x}_{2}=\;-h/2$)	0.724	0.721	% 0.42
$\sigma_{33}$ (MPa) ${(x}_{2}=\;-h/2$)	0.301	0.307	% 1.95

**Table 3:** Midpoint deflection values w of the laminated composite plate with periodic curvature for $E_{2}/E_{1}=10$, as functions of ε and $l_{1}/l_{3}$, based on Reissner–Mindlin (RM) theory and compared with Kirchhoff–Love (KL) theory (×10^−3^ mm).

ε	$l_{1}/l_{3}=$ 0.1		$l_{1}/l_{3}=$ 0.5		$l_{1}/l_{3}=$ 1.0		$l_{1}/l_{3}=$ 2.0	
	RM	KL	RM	KL	RM	KL	RM	KL
0.000	−15.6400	−14.6900	−13.5300	−12.7100	−6.0920	−5.7430	−0.8691	−0.7942
0.001	−15.6800	−14.7100	−13.5500	−12.7200	−6.0960	−5.7460	−0.8692	−0.7943
0.002	−15.7700	−14.7700	−13.6200	−12.7700	−6.1100	−5.7560	−0.8694	−0.7945
0.003	−15.8800	−14.8800	−13.6900	−12.8400	−6.1230	−5.7720	−0.8697	−0.7949
0.004	−16.0700	−15.0300	−13.8300	−12.9500	−6.1490	−5.7940	−0.8703	−0.7955
0.005	−16.5000	−15.2400	−14.1300	−13.1000	−6.2050	−5.8240	−0.8714	−0.7963
0.006	−16.6300	−15.5000	−14.2300	−13.2800	−6.2170	−5.8610	−0.8716	−0.7972
0.007	−16.8700	−15.8300	−14.4000	−13.5100	−6.2470	−5.9070	−0.8722	−0.7983
0.008	−17.9600	−16.2300	−15.1300	−13.7900	−6.3780	−5.9610	−0.8751	−0.7996
0.009	−18.1300	−16.7300	−15.2300	−14.1300	−6.3840	−6.0250	−0.8757	−0.8011

**Table 4:** Midpoint bending moment values of the laminated composite plate with periodic curvature for $E_{2}/E_{1}=10$, as functions of ε and $l_{1}/l_{3}$, based on Reissner–Mindlin theory and compared with Kirchhoff–Love (KL) theory (×10^−2^ kN·m).

ε		$l_{1}/l_{3}=$ 0.1		0.5		1.0		2.0	
		RM	KL	RM	KL	RM	KL	RM	KL
0.000	$M_{11}$	134.4500	134.5500	120.2900	120.7100	61.9464	63.2392	12.2674	12.5752
	$M_{33}$	37.5994	37.4866	51.3074	50.1994	64.3843	63.2189	30.1124	30.1792
0.001		134.7500	134.6900	120.5200	120.8000	61.9979	63.2510	12.2677	12.5730
		37.6800	37.5208	51.4126	50.2534	64.4358	63.2493	30.1159	30.1815
0.002		135.6500	135.0900	121.2100	121.0900	62.1523	63.2866	12.2681	12.5665
		37.9248	37.5335	51.7319	50.4166	64.5905	63.3408	30.1261	30.1883
0.003		136.6100	135.7700	121.9400	121.5700	62.3027	63.3472	12.2682	12.5556
		38.1851	37.8249	52.0766	50.6935	64.7444	63.4951	30.1366	30.1996
0.004		138.3900	136.7600	123.3100	122.2600	62.6069	63.4343	12.2739	12.5403
		38.6687	38.1010	52.7136	51.0911	65.0460	63.7147	30.1590	30.2157
0.005		142.3400	138.0800	126.2300	123.1800	63.2719	63.5506	12.2874	12.5207
		39.7395	38.4704	54.0614	51.6202	65.6921	64.0035	30.2040	30.2366
0.006		143.5900	139.7900	127.1600	124.3600	63.3948	63.6992	12.2831	12.4967
		40.0772	38.9449	54.5115	52.2956	65.8336	64.3665	30.2130	30.2626
0.007		145.8100	141.9300	128.8200	125.8400	63.7057	63.8845	12.2753	12.4683
		40.6795	39.5408	55.3011	53.1370	66.1708	64.8102	30.2365	30.2940
0.008		155.9700	144.5900	136.0500	127.6500	65.3183	64.1115	12.3398	12.4356
		43.4211	40.2799	58.6235	54.1701	67.6906	65.3427	30.3510	30.3398
0.009		157.6500	147.8700	137.0900	129.8700	65.4374	64.3869	12.3963	12.3985
		43.86861	41.1912	59.1295	55.4288	67.7900	65.9741	30.3909	30.3740

**Table 5:** Stress values at $x_{2}=-h/2$ of the laminated composite plate with periodic curvature for $E_{2}/E_{1}=10$, as functions of ε and $l_{1}/l_{3}$, based on Reissner–Mindlin theory and compared with Kirchhoff–Love (KL) theory (×10^−2^ MPa).

ε		$l_{1}/l_{3}=$ 0.1		0.5		1.0		2.0	
		RM	KL	RM	KL	RM	KL	RM	KL
0.000	$\sigma_{11}$	80.6689	80.7320	72.1751	72.4250	37.1678	37.9440	7.3604	7.5451
	$\left(l_{1}/2,l_{3}/2\right)$								
	$\sigma_{33}$	22.5596	22.4900	30.7844	30.1200	38.6306	37.9310	18.0674	18.1080
	$\left(l_{1}/2,l_{3}/2\right)$								
	$\sigma_{23}$	65.9466	0.0000	111.5388	0.0000	84.9880	0.0000	23.9137	0.0000
	$\left(l_{1}/2,l_{3}\right)$								
	$\sigma_{13}$	6.7500	3.2803	11.5788	5.6709	8.9178	4.4281	2.8150	1.4185
	$\left(l_{1},l_{3}\right)$								
	$\sigma_{12}$	70.6984	0.0000	61.2810	0.0000	28.6031	0.0000	5.2043	0.0000
	$\left(l_{1},l_{3}/2\right)$								
		80.8472	80.8110	72.3111	72.4810	37.1987	37.9510	7.3606	7.5438
		22.6080	22.5120	30.8476	30.1520	38.6615	37.9500	18.0695	18.1090
0.001		66.0732	0.0000	111.6988	0.0000	85.0477	0.0000	23.9161	0.0000
		6.7326	3.2850	11.6152	5.6772	8.9752	4.4309	2.8410	1.4188
		70.8415	0.0000	61.3847	0.0000	28.6237	0.0000	5.2050	0.0000
		81.3880	81.0530	72.7233	72.6100	37.2914	37.9720	7.3608	7.5399
		22.7549	22.5800	31.0391	30.2500	38.7543	38.0040	18.0756	18.1130
0.002		66.4577	0.0000	112.1839	0.0000	85.2270	0.0000	23.9232	0.0000
		6.7365	3.2992	11.6886	5.6960	9.0539	4.4394	2.8723	1.4197
		71.2758	0.0000	61.6992	0.0000	28.6858	0.0000	5.2072	0.0000
		81.9631	81.4640	73.1635	72.9400	37.3816	38.0080	7.3608	7.5333
		22.9111	22.6950	31.2459	30.4160	38.8467	38.0970	18.0819	18.1200
0.003		66.8692	0.0000	112.7008	0.0000	85.4041	0.0000	23.9299	0.0000
		6.7010	3.3234	11.7806	5.7277	9.1873	4.4538	2.9332	1.4212
		71.7367	0.0000	62.0356	0.0000	28.7466	0.0000	5.2091	0.0000
		83.0315	82.0570	73.9850	73.3550	37.5641	38.0610	7.3643	7.5242
		23.2012	22.8610	31.6282	30.6550	39.0276	38.2290	18.0953	18.1290
0.004		67.6295	0.0000	113.6625	0.0000	85.7514	0.0000	23.9447	0.0000
		6.7274	3.3581	11.9086	5.7732	9.3038	4.4741	2.9768	1.4233
		72.5879	0.0000	62.6575	0.0000	28.8650	0.0000	5.2120	0.0000
		85.4049	82.8510	75.7376	73.9090	37.9632	38.1300	7.3724	7.5124
		23.8437	23.0820	32.4368	30.9720	39.4152	38.4020	18.1224	18.1420
0.005		69.2805	0.0000	115.7061	0.0000	86.4979	0.0000	23.9760	0.0000
		6.9226	3.4042	12.1213	5.8335	9.3629	4.5008	2.9692	1.4259
		74.4768	0.0000	63.9770	0.0000	29.1199	0.0000	5.2209	0.0000
		86.1532	83.8720	76.2932	74.6170	38.0369	38.2200	7.3698	7.4980
		24.0463	23.3670	32.7069	31.3770	39.5001	38.6200	18.1277	18.1580
0.006		69.8178	0.0000	116.3545	0.0000	86.6569	0.0000	23.9798	0.0000
		6.8299	3.4631	12.2527	5.9099	9.5848	4.5342	3.0745	1.4293
		75.0745	0.0000	64.4056	0.0000	29.1738	0.0000	5.2217	0.0000
		87.4848	85.1570	77.2907	75.5020	38.2233	38.3310	7.3651	7.4810
		24.4077	23.7240	33.1806	31.8820	39.7025	38.8860	18.1419	18.1760
		70.7762	0.0000	117.5433	0.0000	87.0485	0.0000	23.9955	0.0000
0.007		6.8450	3.5365	12.4177	6.0044	9.7489	4.5748	3.1368	1.4332
		76.1572	0.0000	65.1843	0.0000	29.3117	0.0000	5.2271	0.0000
		93.5837	86.7530	81.6303	76.5900	39.1910	38.4670	7.4038	7.4614
		26.0527	24.1680	35.1741	32.5020	40.6144	39.2060	18.2105	18.1990
0.008		74.9249	0.0000	122.5468	0.0000	88.7961	0.0000	24.0731	0.0000
		7.3478	3.6266	12.9149	6.1197	9.8404	4.6232	3.0966	1.4378
		80.9610	0.0000	68.4039	0.0000	29.8974	0.0000	5.2427	0.0000
		94.5901	88.7250	82.2524	77.9200	39.2624	38.6320	7.4377	7.4391
		26.3212	24.7150	35.4777	33.2570	40.6740	39.5840	18.2345	18.2240
0.009		75.5605	0.0000	123.2243	0.0000	88.8829	0.0000	24.0895	0.0000
		7.1691	3.7367	13.0732	6.2588	10.1631	4.6803	3.2484	1.4431
		81.6932	0.0000	68.8359	0.0000	29.9108	0.0000	5.2395	0.0000

## Data Availability

The original contributions presented in this study are included in the article. Further inquiries can be directed to the corresponding author.

## Abbreviations

The following abbreviations are used in this manuscript:

FEM	Finite Element Method
RM	Reissner–Mindlin theory
KL	Kirchhoff–Love theory
MATLAB	Matrix Laboratory
